# Evaluating the cytotoxicity of Ge–Sb–Se chalcogenide glass optical fibres on 3T3 mouse fibroblasts†

**DOI:** 10.1039/d0ra00353k

**Published:** 2021-02-25

**Authors:** David Mabwa, Teo Kubiena, Harriet Parnell, Rong Su, David Furniss, Zhuoqi Tang, Richard Leach, Trevor M. Benson, Colin A. Scotchford, Angela B. Seddon

**Affiliations:** Mid-Infrared Photonics Group, George Green Institute for Electromagnetic Research Group, Faculty of Engineering, University of Nottingham Nottingham NG7 2RD UK david.mabwa@nottingham.ac.uk angela.seddon@nottinghan.ac.uk; Advanced Materials Research Group, Faculty of Engineering, University of Nottingham Nottingham NG7 2RD UK; Manufacturing Metrology Team, Faculty of Engineering, University of Nottingham Nottingham NG7 2RD UK

## Abstract

*In vivo* cancer detection based on the mid-infrared molecular fingerprint of tissue is promising for the fast diagnosis and treatment of suspected cancer patients. Few materials are mid-infrared transmissive, even fewer, which can be converted into functional, low-loss optical fibres for *in vivo* non-invasive testing. Chalcogenide-based glass optical fibres are, however, one of the few. These glasses are transmissive in the mid-infrared and are currently under development for use in molecular sensing devices. The cytotoxicity of these materials is however unknown. The cytotoxicity of Ge–Sb–Se chalcogenide optical glass fibres on 3T3 mouse fibroblast cells is here investigated. Fibres exposed to four different pre-treatment conditions are used: as-drawn (AD), propylamine-etched (PE), oxidised-and-washed (OW) and oxidised (Ox). To achieve the latter two conditions, fibres are treated with H_2_O_2_(aqueous (aq.)) and dried to produce a surface oxide layer; this is either washed off (OW) or left on the glass surface (Ox). Cellular response is investigated *via* 3 day elution and 14 day direct contact trials. The concentration of the metalloids (Ge, Sb and Se) in each leachate was measured *via* inductively coupled plasma mass spectrometry. Cell viability is assessed using the neutral red assay and scanning electron microscopy. The concentration of Ge, Sb and Se ions after a 3 day dissolution was as follows. In AD leachates, Ge: 0.40 mg L^−1^, Sb: 0.17 mg L^−1^, and Se: 0.06 mg L^−1^. In PE leachates, Ge: 0.22 mg L^−1^, Sb: 0.15 mg L^−1^, and Se: 0.02 mg L^−1^. In Ox leachates, Ge: 823.8 mg L^−1^, Sb: 2586.6 mg L^−1^, and Se: 3750 mg L^−1^. Direct contact trials show confluent cell layers on AD, PE and OW fibres after 14 days, while no cells are observed on the Ox surfaces. A >50% cell viability is observed in AD, PE and OW eluates after 3 days, when compared with Ox eluates (<10% cell viability). Toxicity in Ox is attributed to the notable pH change, from neutral pH 7.49 to acidic pH 2.44, that takes place on dissolution of the surface oxide layer in the growth media. We conclude, as-prepared Ge–Sb–Se glasses are cytocompatible and toxicity arises when an oxide layer is forced to develop on the glass surface.

## Abstract 2

We present a study that aims to evaluate the cytotoxicity of Ge_20_Sb10Se_70_ at% glass optical fibres on 3T3 mouse fibroblast cells. To observe the toxicity of these optical fibres, 3T3 fibroblast proliferation was investigated.

## Graphical abstract

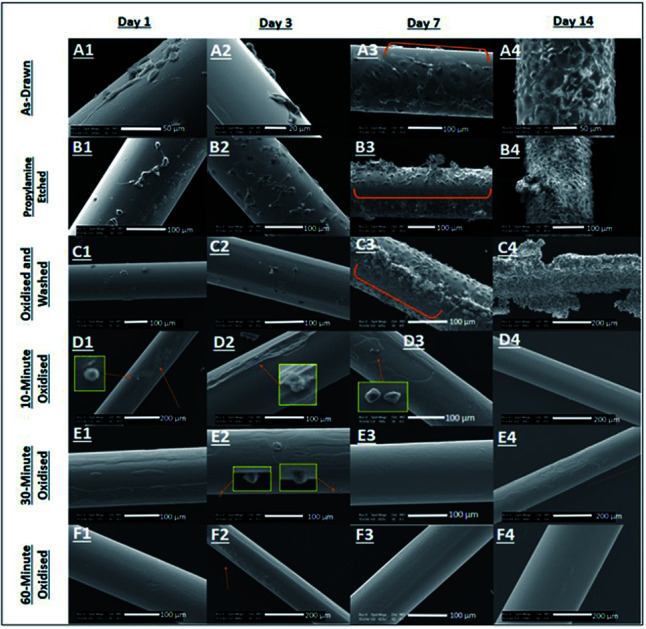

## 1. Introduction

Over 100 different types of cancers occur in humans, and only four: breast, colon, prostate, and lung, are responsible for more than half of all diagnoses, with a comparable proportion of deaths. Cancer diagnosis has however improved in recent decades, with advancements occurring in for example, genetic profiling and diagnostic imaging. A relatively novel method is mid-infrared hyperspectral imaging.^1^ This is based on utilising mid-infrared (MIR) radiation to identify the presence and distribution of chemical species in biological tissue. When a tissue sample absorbs radiation in the MIR region (3.0 μm to 50 μm)^2^ covalently bonded bio-molecular species vibrate to give absorption bands whose absorption and characteristic wavelength, provide information pertaining to the quantity, and nature, of their bonding, respectively.^3^ The rapidity and high diagnostic accuracy of MIR hyperspectral imaging has been demonstrated by many, for instance: Old *et al.*^4^ and Pilling *et al.*^5^ Old *et al.* collected and rapidly processed 45 MIR hyperspectral images of oesophageal tissue (5 minutes per image), then accurately identified neoplastic Barrett's oesophagus with 95.6% sensitivity and 86.4% specificity.^4^ Similarly, Pilling *et al.* collected MIR spectra from 207-breast cancer patients using quantum cascade laser (QCL) imaging (5.55 μm–10.86 μm) and accurately differentiated between malignant and non-malignant stroma with 93.56% specificity.^5^

Along with its numerous benefits, MIR hyperspectral imaging also comes with limitations. It is currently limited to only imaging excised tissue. This is due to traditionally weak MIR light blackbody sources and the lack of optical fibre that transmits sufficiently into the MIR spectral region.^6^ The latter limitation has since been resolved since the advent of chalcogenide-based optical fibres, derived from the chalcogen elements of group 16 in the periodic table (S, Se and Te – excluding O).^7^ With the aim of providing point-of-care testing, in previous work, we developed low optical loss Ge_20_Sb_10_Se_70_ atomic (at) % glass optical fibres capable of transmitting MIR light from 2.5 μm–13 μm.^8^ This glass system opens the possibility of utilising MIR hyperspectral imaging for the diagnosis of cancerous tissue, *in vivo*, through its incorporation into endoscopic probes.^9^

The metalloid elements used in formulating these optical fibres warrant an analysis of their cytotoxicity, prior to their application in point-of-care testing. Similar work was conducted by Wilhelm *et al.*,^10^ who investigated cell coverage by A549 human lung cells on glass preforms of Te_2_As_3_Se_5_ (TAS) at%. This study reported inconsistent and non-reproducible cell-attachment results, a problem attributed by the authors to the presence of arsenic (As) in the glass and a thin surface oxide layer. The toxic behaviour of the TAS glasses was then assessed by submerging the TAS fibres in cell cultures and analysing the cell metabolic activity *via* a WST-1 colorimetric assay. It was concluded that, after the TAS fibres were washed in water for 24 hours, they produced no toxic effect towards the cells (95% to 103% cell viability), whereas unwashed fibres produced a more toxic effect in comparison (65% to 69% cell viability).

As is included in many chalcogenide glass formulations due to its ability to stabilise the covalent glass network. However, the inclusion of this element into medical instruments (*i.e.*, endoscopic probes) raises significant concerns. This is because, even in micro-quantities, As is highly toxic and possesses the potential to irreversibly damage cells and tissue.^10^ In previous work,^11^ we replaced As with antimony (Sb) to produce a glass composition more suitable for biological applications. The toxicity of these Sb-containing compositions has however, not previously been evaluated. This forms the aim of this paper. To produce an in-depth evaluation on the cytotoxicity of Sb-containing chalcogenide glasses and assess whether Sb-based compositions would be viable for their application into medical devices such as endoscopic probes.

Data related to the toxicity of antimony in humans come primarily from studies that investigate its connection with industrial-atmospheric exposure. Potkonjak and Pavlovich^12^ examined the respiratory condition of 51 workers in a Yugoslavian antimony smelter with chest X-ray analysis. X-ray changes indicated antimoniosis. The workers were exposed to 17 mg m^−3^ to 86 mg m^−3^ antimony dust for 9 to 31 years. Antimony trioxide (Sb_2_O_3_) made up 40% to 90% of the dust, antimony pentoxide (Sb_2_O_5_) 2% to 8% and free silica 1% to 5%. Positive X-ray findings characterised antimoniosis as diffuse, densely distributed punctate opacities, irregular in shape, and with a diameter less than 1 mm in the lung. In addition, chronic coughing was reported in 60.8% of workers, chronic bronchitis in 37.2%, chronic emphysema in 34.5%, inactive tuberculosis in 18.2% and pleural adhesions in 27.3%.

The chemo-toxicological characteristics of arsenic and antimony are very similar.^13^ In the trivalent state, both behave clastogenically *in vivo* and *in vitro*, are not directly mutagenic, and have a carcinogenic potential.^14,15^ Genotoxicity of antimony has been shown to be valence dependant but the underlying mechanism for its genotoxicity remains unclear.^14,16^ Kuroda *et al.*^17^ used the sister chromatid exchange (SCE) assay to show that Sb_2_O_3_ at a concentration of 0.34 μL mL^−1^ induced significant genotoxic activity, whereas Sb_2_O_5_ at a concentration of 40 μL mL^−1^ did not. A biomonitoring study by Cavallo *et al.*^18^ evaluated genotoxicity in 23 workers exposed to Sb_2_O_3_ (*via* skin contact and the respiratory pathway) using SCE assay, micronucleus tests and (Fpg)-modified comet assay. The results suggested that Sb(iii) toxicity results from oxidative DNA damage. It has also been suggested by Grosskopf *et al.*^16^ that Sb(iii) interferes with proteins involved in nucleotide excision repair, resulting in the partial retardation of this pathway and an indirect mechanism in the genotoxicity of Sb(iii).

In view of this, we present a study that aims to evaluate the cytotoxicity of Ge_20_Sb_10_Se_70_ at% glass optical fibres on 3T3 mouse fibroblast cells. To observe the toxicity of the fibres, 3T3 fibroblast proliferation was investigated. This involved two trials. Firstly, cells were subjected to elution trials, in which the effect of any glass-derived leachates on the proliferation of the cells was assessed. The concentration of each metalloid species (leachates) was measured, by exposing glass fibres to deionised water over a 14 day period. This allowed for further evaluation of the cell viability outcome. This was followed by direct contact trials, which were performed to observe the adhesion/attachment behaviour of cells on the surface of the fibre. Cell viability was assessed using the neutral red assay; this chemo-sensitive assay was based on the ability of viable cells to incorporate and bind to the supravital neutral red dye. Since genotoxic activity is observed in literature as a response to Sb_2_O_3_, we generated oxides at the glass surface by exposing the Ge_20_Sb_10_Se_70_ at% glass optical fibres to aqueous H_2_O_2_, (H_2_O_2(aq.)_) and the potential toxicity of the oxide produced on 3T3 fibroblasts was evaluated.

## 2. Materials and methods

### 2.1. Ge_20_Sb_10_Se_70_ at% glass optical fibre pre-treatments and sample codes

Ge_20_Sb_10_Se_70_ at% bulk glass rod was synthesised in high purity, using 99.999% purity elements, in a pre-purified silica ampoule, under a 10^−6^ Pa vacuum at 900 °C for 12 h. The bulk glass preform was then drawn into a fibre with a diameter of 210 μm ± 20 μm. More details of the melting procedure can be found in the ESI.† The Ge_20_Sb_10_Se_70_ at% fibres were cleaved using a ruby-tipped scribe (S90R, Thorlabs) under ambient conditions into 5 mm and 10 mm lengths (for direct contact and elution trials respectively) and were subjected to one of three different post-fibre-drawing pre-treatments, as indicated below. All the pre-treatments were conducted under ambient conditions.

#### 2.1.1. As-drawn (AD) and propylamine etched (PE) fibre

As-drawn (AD) fibre pieces were cleaved as described above, then washed in triplicate with acetone (Fisher Chemicals, 99.5%) and isopropanol (Fisher Chemicals, 99.5%). During each wash, the fibres were placed in a 10 mL scintillation vial and held in an ultrasonic cleaner for 5 min. Following this, the fibres were left in ambient conditions to allow the solvent to evaporate then wrapped in lens tissue (Ted Pella, Inc.) and stored in a silica glass vial for up to 5 days before use.

To prepare propylamine etched (PE) fibres, as-drawn cleaved fibre pieces were collectively submerged in 5 mL of propylamine (Sigma-Aldrich, 99.0%), within a scintillation vial for 0.5 h, then washed in triplicate with acetone, and isopropanol. During each wash, the fibres were placed in a 10 mL scintillation vial and held in an ultrasonic cleaner for 5 min. Following this, the fibres were left in ambient conditions to allow the solvent to evaporate, then placed into a new scintillation vial, wrapped in lens tissue, and transferred into an MBraun glove box, with a nitrogen atmosphere, with 0.6 ppm H_2_O and 0.1 ppm O_2_ for storage up to 5 days before use.

#### 2.1.2. H_2_O_2_ aq. treated fibres: O10, O30, and O60

As-drawn, cleaved fibre samples were initially triplicate washed in acetone and isopropanol then submerged in 30% w/v aqueous hydrogen peroxide (H_2_O_2(aq.)_) (Fisher Scientific) for 10, 30 and 60 min. After the timed submersion, the H_2_O_2(aq.)_ was decanted from the fibres, that then were dried for 12 h under dust cover, in ambient conditions. After this process, a white, partially soluble glass surface oxide had formed.^19^

#### 2.1.3. H_2_O_2_ aq. treatment followed by washing of fibres: OW

As-drawn, cleaved fibre samples initially underwent the same pre-treatment as the O60 fibres (see Section 2.1.2). In the same way, a white oxide formed on all the fibre surfaces. However, the fibres then underwent further treatment as follows. Triplicate washing in acetone and isopropanol was carried out, while in an ultrasonic cleaner for 10 min (as described in Section 2.1.1), and this was observed to remove the white surface layer from the fibres.

#### 2.1.4. Further pre-treatment of fibres

For all conditions mentioned above (see Sections 2.1.1 to 2.1.3), fibres intended for direct contact trials were individually solvent washed, allowed to dry and wrapped in lens tissue to prevent ongoing physical damage to the fibre surfaces, whereas fibres intended for eluate production were collectively solvent washed, allowed to dry and wrapped in lens tissue.

After the pre-treatments described in Sections 2.1.1, 2.1.2 and 2.1.3, all fibre samples intended for direct contact and elution trials were disinfected *via* ultraviolet (UV) sterilisation in a class II microbiological safety cabinet (Nuaire biological safety cabinets, Triple Red Ltd) for 90 min, under ambient atmosphere and temperature, before the trials began.

In order to prepare the elution medium (eluate) for each condition, fibre samples from each condition were respectively submerged in culture medium (production method described in ESI†) for 24 h or 72 h, while being horizontally rotated in a sterile 20 mL disposable scintillation vial, at 50 rpm (revolutions per minute, Denley Spiramix 5). Oxidised samples (OW, O30 and O60) were only submerged for 24 h. Different submersion times were investigated to see what effect a longer fibre-matrix interaction time would have on cell viability. After 24 h or 72 h, the culture medium, now the elution medium (eluate) was decanted from the fibres and diluted. The following dilutions by volume of the eluate were then produced using the culture medium as the diluent: 100%, 75%, 50%, and 25%. The diluents were placed into 5 mL sterile universal tubes and stored in a chemical refrigerator (for a maximum of 3 days) at 4 °C until needed, after which the diluents were warmed to 37 °C in a water bath and used.

The control sample used in this work was tissue culture plastic (TCP), *i.e.* an untreated, fibreless well of a 96-well plate (material: polystyrene, ThermoScientific).

### 2.2. Cytotoxicity tests

#### 2.2.1. Elution

For the elution trials, the as-drawn, Ge_20_Sb_10_Se_70_ at% optical fibres were cleaved and prepared to satisfy the ISO standard (10993-12:2012) of 6 cm^2^ of any solid material, with a thickness less than 0.5 mm, per mL of liquid.^20^ In this trial, a total of 31.05 mL of culture medium was used as the elution medium, with 186.3 cm^2^ of fibre surface area. Cells were seeded onto 5 wells for each of the seven different conditions (*viz.*: AD-24 h, AD-72 h, PE-24 h, PE-72 h, OW, O60 and O30) in a 96-well plate at a cell seeding density of 15 × 10^3^ cm^−2^ and incubated (Nuaire DH Autoflow CO_2_ Air-Jacketed Incubator) at 37 °C with 5 vol% CO_2_, for 24 h, to allow cells to attach to the bottom of each well. After the 24 h incubation period, the appropriate eluate was added to each well as above described, and cell viability assessed *via* the neutral red assay at day-1 and day-3, after the addition of the eluate (this counting of days, as *e.g.* day 1, excludes the prior 24 h incubation period). Control 3T3 fibroblast samples were grown on the same well plate, however using unmodified culture medium (described in ESI†).

#### 2.2.2. Direct contact

Following the pre-treatments, indicated in the sub-sections of Section 2.1, seven fibre pieces (each of 5 mm length) from each pre-treatment condition were selected and transferred into a new 96-well plate (1 fibre piece per well), so that the sample number of fibre pieces for each pre-treatment condition was 5, giving 35 fibre samples in all. Two additional pre-treated fibre pieces were added to the 96-well plate per pre-treatment condition (*i.e.* for each of: AD, PE, OW, O10, O30, O60) totalling now 49 fibre samples in all; the new fibre pieces were added specifically to enable their later removal directly for scanning electron microscopic (SEM) observation. These additional fibres were added due to the destructive nature of the neutral red dye; cells that have absorbed the dye, rupture during the destaining process^21^ and so, in order to observe the intrinsic growth pattern of the cells, these two additional fibres per pre-treatment condition were used, which would not undergo neutral red staining. Cells were then seeded onto all 49 fibres in the 96-well plate at a seeding density of 12.4 × 10^3^ cm^−2^. Cell viability was assessed *via* the neutral red assay, for those fibre samples not destined for SEM observation, at day 1, 3, 7 and 14. Cell viability associated with each type of fibre pre-treatment condition at each time point was also compared to the control samples (*n* = 5) grown on TCP.

#### 2.2.3. Cell viability after direct contact and elution trials *via* the neutral red assay

To quantify the cell viability after direct cell contact with the fibres and the elution trials, the neutral red assay was performed. The method for this was as follows. 20 mL of neutral red stock was prepared in a 4 mg to 1 mL ratio of neutral red dye to deionised water in a 25 mL sterile universal tube (Sterilin, UK), and then protected from photocatalytic degradation by wrapping in aluminium foil (Terinex). Neutral red medium was then prepared in a 1 : 100 (neutral red stock : culture media) dilution under sterile conditions, centrifuged at 1200 rpm for 4 minutes so that any crystals that may have formed, would settle at the base of the 25 mL sterile universal tube; care was taken not to shake the tube, so as not to re-suspend the crystals. The neutral red medium was not stored before/after an experiment. Any excess was discarded.

At the indicated time points (day-1, day-3, day-7, and day-14 for direct contact trials and day-1 and day-3 for elution trials), the cells were submerged in neutral red medium and incubated for 2 hours at 37 °C with 5 vol% CO_2_. After incubation, the unincorporated neutral red was decanted and discarded. The cells were then washed in triplicate with phosphate buffer saline (PBS, Oxoid UK). Following the triplicate washes with PBS, a destaining solution was added to each well, to extract the incorporated dye from the viable cells. The destaining solution was prepared by volume in ambient temperature, by mixing pure ethanol (Scientific Laboratory Supplies, 99%), with deionised water and aqueous glacial acetic acid (99.74%, Fisher Scientific, UK)) to a ratio of 5 : 4.9 : 0.1, respectively. The 96-well plate was then placed on a plate shaker (Titramax 100, Heidolph) at 300 rpm for 10 min under ambient conditions to extract the incorporated neutral red dye from the cells. Finally, the 96-well plate was placed in the BioTek™ ELx800™ UV (Fisher Scientific) plate reader (primary wavelength 490 nm, reference wavelength 630 nm), to quantify the neutral red dye concentration. Cell viability was expressed as a percentage of the control of which, a cell viability under 70% of the viability expressed by the control cells, indicated material cytotoxicity, as stated by the ISO standards ISO 10993-5:2009.^22^

#### 2.2.4. Chemical analysis: Ge, Sb, and Se concentration in leachates – *via* – inductively coupled plasma mass spectrometry (ICP-MS)

Ge, Sb, and Se concentrations (mg L^−1^) in aqueous leachates, after exposure to AD, PE and O60 were measured using the single quadrupole inductively coupled plasma mass spectrometer (ICP-MS) iCAPQ. For these leaching experiments, fibre samples from each condition were respectively submerged into deionised water at 37 °C for 1, 3, 7 or 14 days, while being horizontally rotated in a sterile 20 mL disposable scintillation vial, at 50 rpm (revolutions per minute, Denley Spiramix 5).

### 2.3. Materials and methods (ESI†)

See ESI for the following details:

(1) Bulk Ge_20_Sb_10_Se_70_ at% preform fabrication and fibre preparation.

(2) A physical analysis of Ge_20_Sb_10_Se_70_ at% glass optical fibres using coherence scanning interferometry and scanning electron microscopy.

(3) A pH physical analysis of the following eluates: AD, PE and O60.

(4) The 3T3 fibroblast cell-line and method for producing the culture medium.

(5) Statistical analysis method performed on the results of this work.

## 3. Results

To recap, to simplify interpretation of the results, as described in Section 2.1, AD, PE, OW, O10, O30 and O60 fibre samples were prepared. Fibres were initially cleaved into 5 mm and 10 mm long lengths (for direct contact and elution trials, respectively), then subjected to one of three pre-treatment conditions. These were, submersion in propylamine for 0.5 h (PE); submersion in H_2_O_2(aq.)_ for 10, 30 and 60 min and subsequent drying in ambient conditions for 12 h, to allow the build-up of a glass oxide layer (O10, O30, O60, respectively) and submersion in H_2_O_2(aq.)_ for 60 min and subsequent drying as previously described and triplicate washing in both acetone and isopropanol to remove the oxide layer (OW) then drying in ambient conditions *via* evaporation. AD fibres were not subjected to any pre-treatment conditions.

### 3.1. Chemical analysis: Ge, Sb and Se concentration in aqueous leachates

**Fig. 1:**
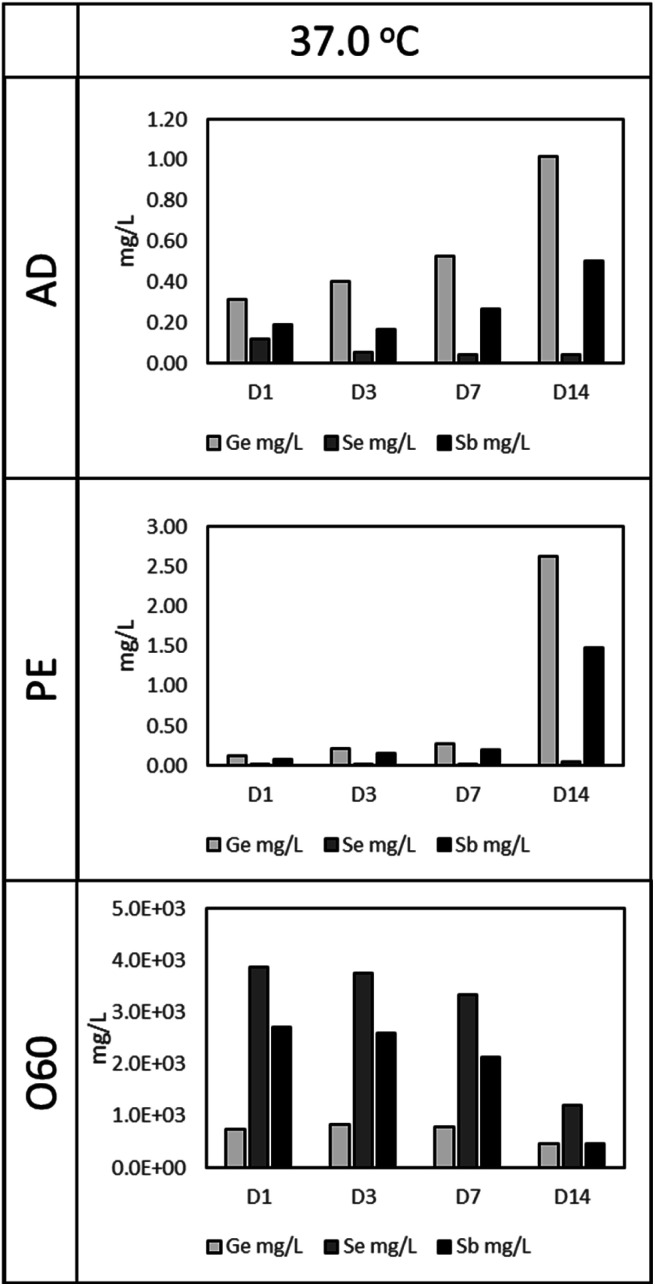
The concentration of Ge, Sb and Se in aqueous leachates after exposure of fibre (conditioned as AD, PE and O60) to deionised water at 37 °C for 1, 3, 7 and 14 days (D1, D3, D7 and D14, respectively).

As in Fig. 1 (37 °C leaching temperature), O60 resulted in the highest Ge, Se, and Sb concentration, compared to AD and PE leachates. The concentration of Se and Sb in O60 dropped as the time for leaching increased, from 3858 mg L^−1^ and 2702 mg L^−1^, respectively in D1 to 1207 mg L^−1^ and 459 mg L^−1^, respectively, at D14. This drop is, however, more notable from D7 to D14, a 175.6% (Se) and 364.8% (Sb) drop, compared to the drop in concentration from D1 to D7 (Se: 16% and Sb: 26.7%). This time dependant relationship with concentration is also observed in AD and PE leachates. In AD, Ge and Sb concentrations increase by 222% and 159%, respectively, from D1 (Ge: 0.31 mg L^−1^, Sb: 0.19) to D14 (Ge: 1.02 mg L^−1^, Sb: 0.50 mg L^−1^), while Se is seen to decrease by 61.7% from D1 (Se: 0.12 mg L^−1^) to D14 (Se: 0.045 mg L^−1^). In PE, Ge and Sb concentrations increase by 1955% and 1757% from D1 (Ge: 0.13 mg L^−1^, Sb: 0.08 mg L^−1^) to D14 (Ge: 2.63 mg L^−1^, Sb: 1.48 mg L^−1^), however, this increase is more prominent from D7 to D14 (Ge: 832% and Sb: 663%), compared to D1 to D7 (Ge: 121% and Sb 143%). Se was observed to increase from 0.017 mg L^−1^, in D1, to 0.052 mg L^−1^ in D14 (a 196.5% increase).

### 3.2. Cell response

#### 3.2.1. Elution

Fibre samples were prepared for elution trials by satisfying the ISO standard (10993-12:2012) of surface area of 6 cm^2^ per mL of liquid for any solid material with a thickness below 0.5 mm,^20^ see Section 2.2.1. To prepare the eluate for each pre-treatment condition, fibres from each condition were separately submerged together in culture medium for 24 h or 72 h, while being horizontally rotated in a scintillation vial at 50 rpm.

**Fig. 2:**
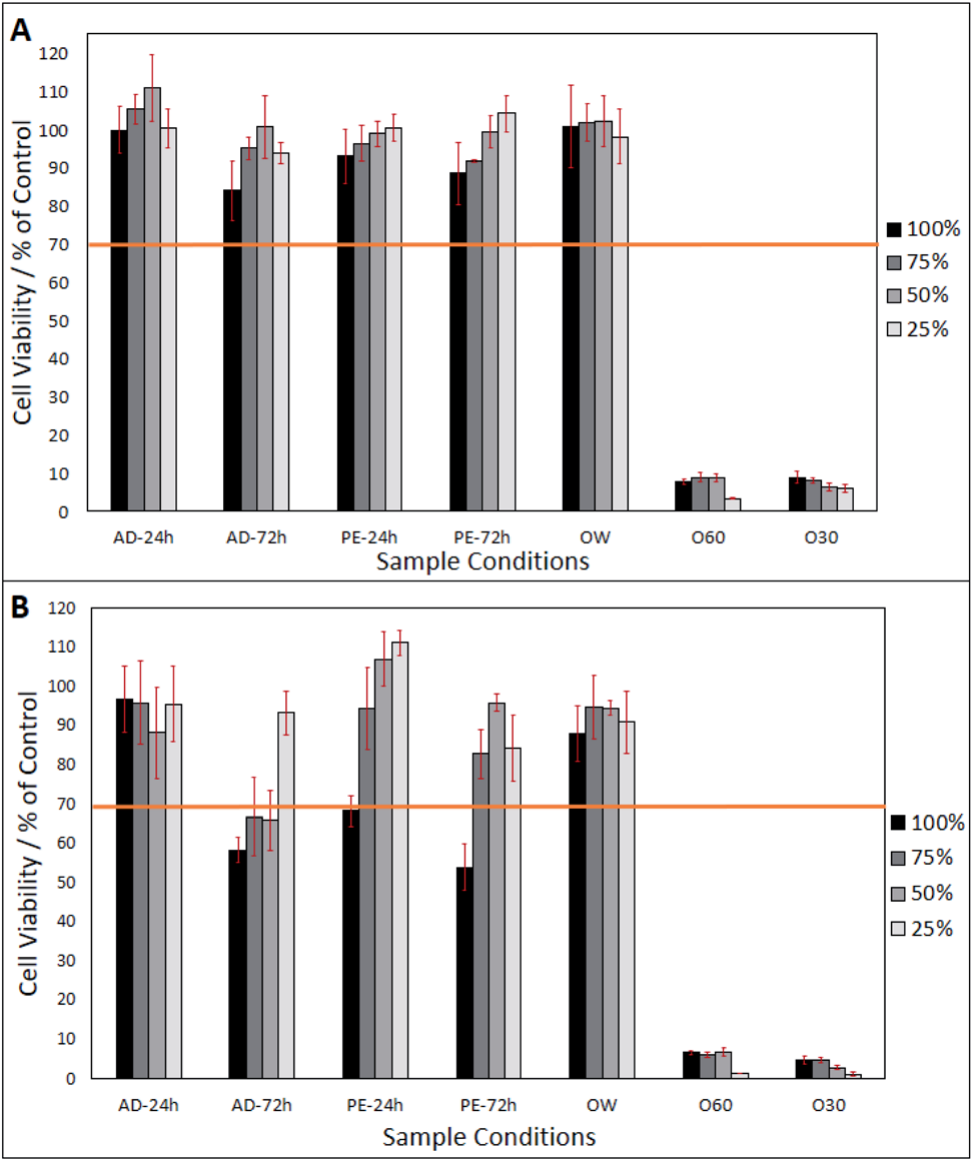
Neutral red assay of cells grown in eluates produced from AD-24 h, AD-72 h, PE-24 h, PE-72 h, OW, O60 and O30 concentrates. (A) Cell viability 24 hours after eluates were added to 3T3 fibroblasts. (B) Cell viability 72 hours after eluates were added. Sample number = *n*5. Significant differences determined using Tukey HSD, multiple comparisons, when *p* = <0.05.

After day-1 (from Fig. 2A), fibroblasts show a viability of >80%, in all eluates, excluding O30 and 060 which only show a viability of <20%. In Fig. 2A, a lower eluate concentration in PE-24 h and PE-72 h is seen to result in higher fibroblast viability. This correlation is also observed in AD-24 h, AD-72 h, and OW, up to an eluate concentration of 50%. At an eluate concentration of 25% in AD-24 h, AD-72 h and OW, the cell viability dropped by 10.4%, 6.8% and 4%, respectively. No statistically significant differences were found between the viabilities of cells grown in AD-24 h, AD-72 h, PE-24 h, PE-72 h and OW. Significant differences were found between the viabilities of cells grown in O60 and O30 and all other eluates, but not between O60 and O30. Fig. 2B shows that cell viability decreased in all eluates, excluding 50% and 25% concentrated PE-24 h, which increased by 8.2% and 10.7%, respectively, when compared to the cell viability observed in PE-24 h after day-1. Cell viability was highest in 25% concentrated PE-24 h (111.1% ± 3.1%) and lowest in 100% concentrated PE-72 h (53.9% ± 5.9%). A notable drop in the pH of O60 was also observed. The pH of the growth media was originally 7.42, and this dropped to 2.44 after 24 h interaction time with O60 fibres. It is inferred that this occurred when glass oxide from the surface of the O60 fibre samples dissolved into the growth media.

#### 3.2.2. Direct contact

**Fig. 3:**
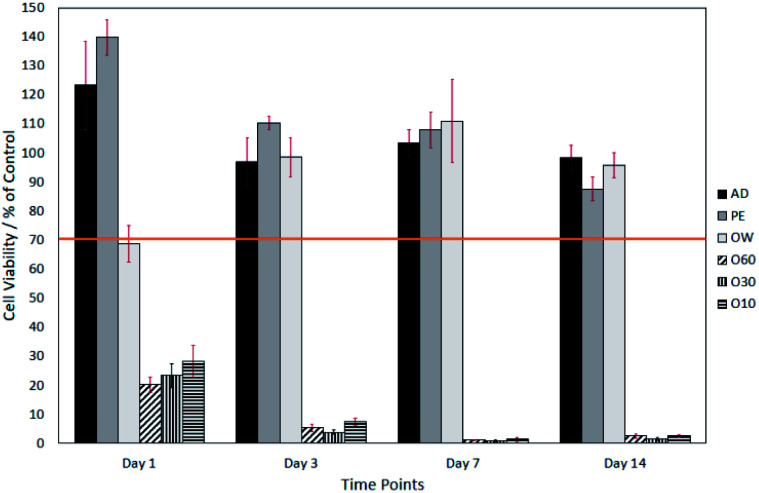
Neutral red assay of cells grown on AD, PE, OW, O60, O30 and O10 surfaces. Cell viability is displayed over a 14 day time period. Sample number = *n*5. Significant differences determined using Tukey HSD, multiple comparisons, when *p* = <0.05.

Fig. 3 shows the viability of fibroblasts over a period of 14 days grown on AD, PE, OW, O60, O30 and O10 fibre surfaces, following direct contact trials as described in Section 2.2.2. Most notable is the significantly lower cell viability on O10, O30 and O60 fibre surfaces from day 1–14. On day-1, cell viability was highest on PE fibre surfaces (139.9% ± 6.1%) and lowest, (not including O10, O30 and O60 fibres) on OW fibre surfaces (68.7% ± 6.2%). Also, on day-1, AD surfaces resulted in a fibroblast cell viability of 123.4% ± 15.2%. On day-3, cell viability on AD and PE fibre surfaces decreased to 92.8% ± 8.2% and 110.2% ± 2.2%, respectively. The fibroblast cell viability on the OW fibre surface, in contrast, had increased by 29.74% to reach 98.5% ± 6.7%. On day-7, a small increase in cell viability on AD (6.6% increase) and OW (12.5% increase) surfaces was recorded rising to *viz.*: 103.4% ± 4.7% and 111.0% ± 14.3%, respectively. A 2.4% decrease in cell viability was recorded on PE surfaces. On day-14, cell viability on AD, PE and OW surfaces had decreased by 5.0%, 20.3% and 15.3% relative to their 7 day viabilities, respectively.

### 3.3. SEM analysis

#### 3.3.1. Day-1

**Fig. 4:**
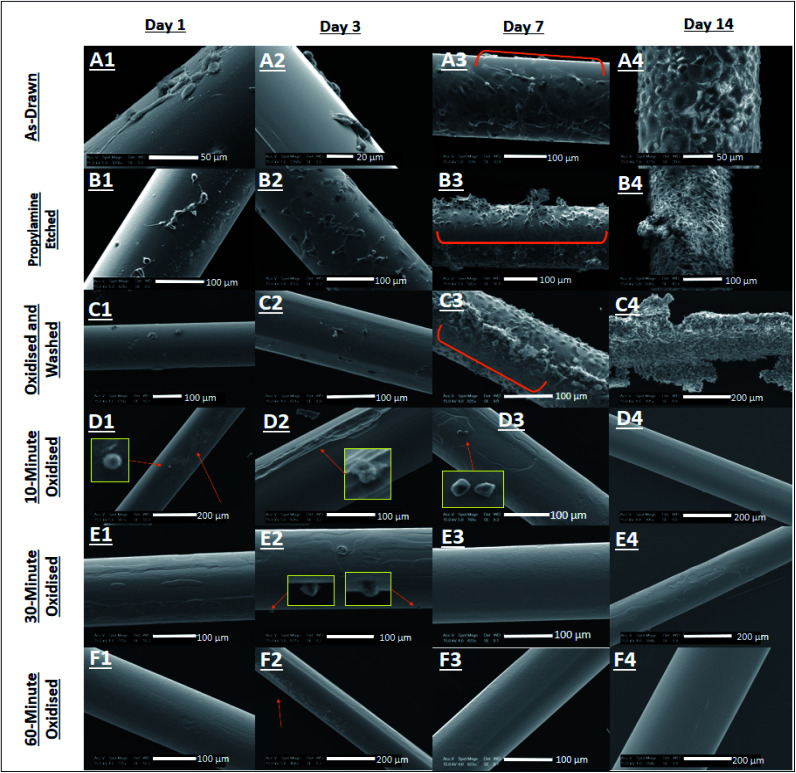
Scanning electron micrographs of cell growth on Ge_20_Sb_10_Se_70_ fibres over a 14 day period. A1–A4 = AD fibre, B1–B4 = PE fibre, C1–C4 = OW fibre, D1–D4 = O10, E1–E4 = O30, F1–F4 = O60 fibre.

On day-1, a very low distribution of fibroblasts was observed on AD, PE and OW surfaces, as seen in Fig. 4A1, B1 and C1, respectively. Cell shape on day-1 ranged from elongated structures with lamellipodia protruding from the surface (Fig. 4A1 and B1) to globular structures possessing no observable lamellipodia (Fig. 4C1). No cells were observed to have adhered on O10, O30 and O60 surfaces at day-1, as seen in Fig. 4D1, E1 and F1. A single cell was however seen to have attached onto the O10 surface (see inset in Fig. 4D1), maintaining a globular structure.

#### 3.3.2. Day-3

On day-3, a very low distribution of fibroblasts was seen on AD and OW surfaces (Fig. 4A2 and C2). A higher distribution of fibroblasts on the PE surface was observed (Fig. 4B2). Cell morphology on PE and OW surfaces ranged from fully spread to globular, whereas only globular cells are observed on the AD surface. Although no extensive attachment of cells was observed on O10, O30 and O60 surfaces (Fig. 4D2, E2 and F2), some structures assumed to be fibroblasts were found still attached to O10 and O30 surfaces (see insets in Fig. 4D2 and E2).

#### 3.3.3. Day-7

By day-7, cell confluency is observed on AD, PE and OW surfaces, as seen in Fig. 4A3, B3 and C3, respectively. Areas on Fig. 4A3, B3 and C3 indicated by orange brackets show the section of fibre that was in contact with the well plate, hence the reduced cell density. No cells were observed on O60, O30 and O10 fibre surfaces in contrast to day-1 and day-3. In Fig. 4D3, indicated by the inset image, are structures found on the fibre surface, not believed to be cells. Due to their crystalline appearance, it is believed that these structures are inorganic crystals developed from interaction of the culture medium with the oxide surface of the fibre.

#### 3.3.4. Day-14

At day-14 the multi-layering of cells was observed on AD, PE, and OW surfaces, see Fig. 4A4, B4 and C4. Moreover, fibroblasts displayed uncontrolled growth on the PE surfaces, as a cell mass was observed to have formed. No cells were observed on O10, O30 and O60 surfaces.

#### 3.3.5. Results (ESI†)

See ESI for the following results:

(1) Surface roughness of the fibres obtained *via* coherence scanning interferometry.

(2) Surface texture obtained *via* SEM.

(3) pH measurements.

(4) Concentration of Ge, Sb, and Se obtained *via* intercoupled plasma mass spectrometry.

## 4. Discussion

In this study, a simple assessment of the cytotoxicity of Ge_20_Sb_10_Se_70_ at% chalcogenide glass optical fibre is presented. AD fibres were tested as they represent optical fibres that could be applied to MIR-based medical devices (*i.e.* endoscopic probes). When exposed to air, a passivating oxide layer begins to form on the surface of chalcogenide-based fibres. Fibres exposed to air for long periods of time (*e.g.* >1 year) can develop a surface oxide layer, with a thickness >30 Å.^19^ It has been previously shown^10^ that, when exposed to air for an extended period of time (3 years), fibres exert a significant toxic effect, however, when these fibred are washed, this toxic effect disappears. PE fibres are a representation of these “washed” fibres. When fibres were exposed to propylamine, this oxide layer was completely stripped off the fibre, and cells are therefore, exposed to ‘bare’ glass and not an oxide layer as in AD.

The fibres used in this study were exposed to air for no longer than 90 days, and so, an oxide layer thick enough to influence cell response, it is suggested, would not have developed. To observe a similar glass-oxide based cytotoxicity as in ref. 10, a surface oxide layer was forced to develop *via* H_2_O_2_ aq. exposure. Finally, the OW treatment was selected to observe any remnant cytotoxicity in fibres, post H_2_O_2_ aq. treatment. In this condition, cells were exposed to fibres that were first treated with H_2_O_2_ aq. then washed to remove the surface oxide layer.

The surface oxide layer in O10–O60 was observed to be partially soluble in the cell growth medium. Once dissolved, this resulted in a notable drop in pH from the physiological 7.49 (ref. 23) to the acidic 2.44. This drop in pH was shown, through direct contact and elution trials, to have a toxic effect on cell viability. The elution trials show that a 1 day exposure to oxidised eluates was enough to result in a cell viability of <10% (see Fig. 2). Direct contact trials also show that a 1 day exposure to fibre surfaces with a glass oxide layer results in a cell viability of <30%, which dropped to <3% after day-3 (see Fig. 3).

This toxic effect on fibroblast cells may be explained as follows. Alterations to the cellular microenvironment can significantly affect cell physiology and induce pathology.^24^ An important aspect of the extracellular environment is its pH. This must be kept within strict boundaries (between 6 and 8 according to ref. 23) to facilitate correct cellular function and prevent cell death.^25,26^ Pathologies associated with an acidic extracellular environment include ischemia (pH <6.3 (ref. 27)), hypercapnia^28^ and metabolic acidosis (pH <7.35 (ref. 29)). Furthermore, mitochondrial dysfunction and the acidification of the extracellular environment have been linked to increased levels of reactive oxygen species (ROS) within cells.^30,31^ ROS (such as, superoxide (O_2_^−^), singlet oxygen (^1^O_2_), hydroxyl radical (OH^.^) and H_2_O_2_) are formed as products under physiological conditions as a result of the partial reduction of O_2_.^32^ Low concentrations of ROS are required to maintain normal physiological functions such as cellular proliferation, signal transduction, immunity, and genetic expression.^32,33^ However, an increased concentration of ROS results in the induction of oxidative stress.^24^

In a study by Teixeira *et al.*^24^ HEK293 cells were used to evaluate the effects of extracellular acidification by ambient temperature HCl aq. addition. They found that lowering the extracellular pH from 7.2 to 5.8, lowered cell viability by 70%, decreased cytosolic pH, hyperpolarised the mitochondrial membrane potential and increased the ROS levels. These results suggest that an acidic extracellular environment can induce cell death through an mPTP opening and ROS-mediated pathogenic pathway. This supports earlier work by Xue and Lucocq^34^ who showed that low extracellular pH results in the activation of the c-Jun N-terminal kinase (JNK) pathway, an important regulator of various cellular processes, including apoptosis, in Swiss 3T3 cells.^35,36^ In our work, the extracellular pH dropped from 7.49 to 2.44, and this was paralleled with a +70% drop in cell viability observed in both elution and direct contact trials after 24 h. Considering former work,^24^ the primary cause of cell death observed in our work is suggested to result from a potential increase in the concentration of ROS within the O10, O30 and O60 eluates.

A secondary potential cause of cytotoxicity involves the possible influence of Ge, Sb and Se metalloid oxides. Metal oxide nanoparticles (MO-NPs) are known to be highly toxic to biological tissue, so much so that some authors have demonstrated their potential applicability in cancer therapy.^25–27,37–39^ These oxide nanoparticles have shown toxic effect *via* pro-apoptotic activity, autophagy, proliferation inhibition, metal ion release and the increase of ROS.^38,40^ In a study by Wilhelm *et al.*^10^ on the biocompatibility of Te–As–Se glass fibres, they observed pronounced toxicity only for fibres exposed to air for 3 years. This toxicity was attributed to the formation of a soluble As_2_O_3_ layer on the fibre surface, which when exposed to aqueous solution, results in the release of arsenic (As) into solution as both As(iii) and As(v). As then exert its toxicity by inactivating vital enzymes involved in ATP generation and DNA synthesis and repair.^41^ Similar results were observed in this study when fibre surface oxidation was chemically enforced over a shorter time. The proliferation rates of fibroblasts exposed to O10, O30 and O60 surfaces and eluates were significantly lower, when compared to the other glass surfaces. As mentioned above, an additional explanation of this is the release of metal ions into the growth media. The question to now address is, which metal oxide is the most plausible cause for this toxicity, GeO_2_, Sb_2_O_3_, Sb_2_O_5_, Se–O or SeO_2_?

It is generally accepted, that germanium metal nanomaterials have low toxicity,^42^ are non-carcinogenic and even inhibitory to the formation of tumour cells.^43,44^ Conversely, as discussed by Lin *et al.*,^45^ inorganic germanium compounds such as GeO_2_, can result in neuro- or nephrotoxicity, but only after very high doses (*e.g.* a minimum oral uptake of 5 g GeO_2_ per day for a 70 kg adult) and long-term exposures. In the same paper the authors showed that fabricated nano-Ge and nano-GeO_2_ are non-toxic to Chinese hamster ovary (CHO) K1 cells, displaying a relative viability greater than 70%. This shows that Ge and its products possess very low toxicity, such that it has found use as a health supplement, in the form of Ge-132.^46^ The levels of Ge observed in our work were considerably lower than the toxic levels indicated in literature (see above). While assessing the concentration of metalloid elements in our leachates *via* ICP-MS, we observed a maximum Ge concentration of 823.8 mg L^−1^, in O60 leachates after 3 days of dissolution, the toxicity of which, is attributed to the acidic extracellular microenvironment, generated by the drop in pH. We also observed a maximum Ge concentration of 1.02 mg L^−1^ and 2.63 mg L^−1^, in AD and PE leachates respectively, after 14 days of dissolution, and no toxicity was observed. GeO_2_ is therefore assumed not to contribute to cell death observed in our work.

Regarding antimony, a weak congruency exists amongst investigators on the toxicity of Sb_2_O_3_. Titma *et al.*^47^ have shown that, when human lung epithelial (A549) cells are exposed to 100 μg mL^−1^ of Sb_2_O_3_, cell viability falls from approximately (approx.) 170% after 12 hours, to approx. 30% after 36 hours. This agrees with the conclusions drawn by Mann *et al.*^48^ and Verdugo *et al.*^49^ that Sb_2_O_3_, Sb(iii) and Sb(v), inhibit cell growth and induce apoptosis, by utilising similar signalling pathways to As_2_O_3_, As(iii) and As(v), resulting in similar toxicities in acute promyelocytic leukaemia (NB4 APL) and human embryonic kidney (HEK-293) cell lines. On the contrary, Omura *et al.*^50^ showed a lack of toxicity resulting from Sb_2_O_3_ on the count, motility and morphology of rat and mouse sperm cells. Even at a repetitive administration of a 1.2 g kg^−1^ dose for 4 weeks, no toxicity was reported in mouse or rat testes. A possible explanation for this discrepancy is that the tissue distribution of Sb_2_O_3_ does not involve the testes, but primarily, the liver and kidneys. To add to the incongruity, Bregoli *et al.*,^51^ within the same study, showed that Sb_2_O_3_ nanoparticles (NPs) demonstrated toxicity towards primary cultures of human hematopoietic progenitor cells but not to human hematopoietic immortalised cell lines. The authors assigned this difference to the tumorigenic alterations of cells lines. In our studies of enforced surface oxidation of Ge–Sb–Se glass optical fibres, we cannot rule out the presence also of antimony pentoxide, however we find no toxicology reports about this particular oxide. As discussed in ref. 52, the human body is primarily exposed to antimony, *via* the inhalation route. Exposure *via* the gastrointestinal (GI) tract is low (<1%) and is limited by the emetic properties of antimony compounds. Finally, uptake through the skin makes no significant contribution to systemic exposure.^52^ The occupational aerosol exposure limit set by the American Conference of Governmental Industrial Hygienists (ACGIH) is 0.5 mg m^−3^.^53^ Cases where exposures are significantly in excess of this (4 mg m^−3^ to 86 mg m^−3^),^12,14^ have been associated with pulmonary toxicity.^12,14,53^ Pustular skin eruptions and antimony dermatosis have also been observed in people working with antimony and antimony salt.^12,14,54^

In our work, Sb ion concentration (see Fig. 1) in AD leachates after leaching in deionised water for 24 h and 72 h elution, at 37 °C (0.194 mg L^−1^ (*i.e.* 194.4 mg m^−3^) and 0.165 mg L^−1^ (*i.e.* 165.2 mg m^−3^), respectively) far exceeded the limits set by the ACGIH (0.5 mg m^−3^). Although elution was in aqueous media, and not in pure deionised water, it is surmised that the eluant Sb concentration would very likely have mirrored the aqueous leachate Sb concentration. It is notable that no cytotoxicity was observed in cells exposed to these eluates. These results seem to contradict Titma *et al.*,^47^ who showed that 100 μg mL^−1^ of Sb_2_O_3_ NPs result in a drop in cell viability from 100% to ∼45% in Balb/c 3T3 cells. These discrepancies are discussed later in this Discussion.

Selenium (Se) is an essential trace element incorporated into selenoproteins as selenocysteine. There are 25 selenium-based proteins in the human selenoproteome, with functions ranging from protection against oxidative damage to the regulation of cellular processes.^14^ Conversely, Se compounds have been shown to possess a clear cytotoxic activity against malignant cells.^55,56^ Forootanfar *et al.*^57^ investigated the cytotoxicity of SeO_2_ and Se NPs (spherical and ranging from 80 nm to 220 nm in size), using the MTT (3-(4,5-dimethylthiazol-2-yl)-2,5-diphenyltetrazolium bromide) assay. They found that the IC_50_ was 6.7 μg mL^−1^ ± 0.8 μg mL^−1^ in the MCF-7 cell-line treated with SeO_2_. Se NPs produced the same effect but at a significantly higher concentration of 41.5 μg mL^−1^ ± 0.9 μg mL^−1^. In previous work by Suzuki *et al.*,^55^ it was concluded that Se compounds induced apoptosis by causing endoplastic reticulum stress and activating the intrinsic apoptotic pathway. We however, observed no cytotoxic effect on cells exposed to AD eluates, containing 0.05 mg L^−1^ (after a 72 h elution time), see Fig. 1.

A tentative explanation for the discrepancies with the literature discussed here lie in the action of Se in high oxidative stress situations, and its interaction with As (and by extension, Sb). Firstly, it has previously been shown^58^ that Se-NPs activate the ATF4, SOD2 and Bcl-xL genes (the anti-oxidative stress pathway), to counter hydroquinone (HQ) induced oxidative stress and protect human dermal fibroblast cells. Additionally, internal ROS species were determined to be lower per cell, in cases where cells were exposed to Se-NPs (during HQ induced stress) in comparison to no exposure. Secondly, some authors have found that Se decreases the toxicity of As.^59,60^ As and Se have similar methylation pathways, and so, can mutually impede the secretion of their methylation metabolite.^61^ This led to the hypothesis that an As–Se compound is formed, that causes less damage on cells than As or Se alone.^62^ This compound was first discovered as seleno Bis(*S*-glutathionyl) arsinium ion [(GS)_2_AsSe]^−^.^63^ It was later found that simultaneous entry of As and Se into a cell, results in the formation of (GS)_2_AsOH first, which is then attacked by HSe^−^, displacing its –OH group, to form [(GS)_2_AsSe]^−^,^64^ which can later be excreted from the cell. Furthermore, Rossman and Uddin,^65^ proposed that Se can guard against As induced oxidative damage through the upregulation of selenoproteins, thioredoxin reductase and glutathione peroxidase. Due to the similar chemical and toxicological characteristics of As and Sb, we suggest that a similar interaction between Se and Sb, forming a seleno–antimony (Sb–Se) detoxication conjugate may have occurred in cells exposed to AD and PE eluates, and so, resulted in the mutual reduction of the toxicity of Sb and Se, and provided increased protection from Sb or Se based oxidative stress. This hypothesis is supported by Feng *et al.*,^66^ who showed that Se alleviated the toxicity of Sb in plants by inhibiting the uptake of Sb and relieving oxidative stress derived from Sb exposure.^67^

The level of Se generated in this study (discounting the levels produced in O60 leachates) is far lower than the permissible exposure limit of Se (400 μg day^−1^).^68^ The level of Sb, produced in this study (discounting levels produced in O60 leachates) is however, higher than the permissible exposure limit set by the ACGIH (0.5 mg m^−3^).^53^ We have however shown that, even when this is the case, there is a lack of cytotoxicity observed from AD fibres (*via* cell adhesion and cell propagation). This demonstrates the potential biocompatibility of medical devices developed using the Ge_20_Sb_10_Se_70_ at% glass composition.

Here, the surface texture of Ge_20_Sb_10_Se_70_ at% optical fibres was modified by etching with propylamine under ambient conditions or through oxidation by H_2_O_2(aq.)_ (see Section 2.1). The results (shown in Fig. S1 and S2 of ESI†) have demonstrated that Ge_20_Sb_10_Se_70_ at% optical fibres are more susceptible to surface damage caused by oxidation by H_2_O_2(aq.)_ for 60 min, than propylamine etching, as surfaces that underwent the former, generated the rougher surfaces (PE Sa = 169 nm ± 15 nm and O60 Sa = 236 nm ± 84 nm), results not shown here. This is not believed to have influenced cell viability, as cells would not have had the time to respond to the altered rougher surface due to the drastically altered chemistry of the cellular microenvironment after exposure to oxidised eluates and fibre surfaces.

## 5. Conclusions

Using a 3T3 fibroblast cell model, we provide evidence that unmodified Ge_20_Sb_10_Se_70_ at% optical fibres do not produce a cytotoxic response. A cytotoxic response was only observed after a glass oxide layer was forced to develop on unmodified, as-annealed, Ge_20_Sb_10_Se_70_ at% optical fibres, through submersion in H_2_O_2(aq.)_. Such oxide growth does not occur under normal ambient conditions, nor on exposure to pH neutral water, but on exposure to a strong oxidising agent like H_2_O_2(aq.)_. The primary cause of toxicity is attributed to the dramatic pH change that occurred in the extracellular environment, after interaction with the glass oxide. This drop in pH from the physiological 7.49 to the acidic 2.44 resulted in a +70% decrease in cell viability after 24 h in both elution and direct contact trials. A secondary negative influence on cell viability is attributed to the presence of Sb_2_O_3_ and SeO_2_ in solution. The lack of cytotoxicity in cells exposed to AD and PE samples is attributed to the mutual antagonistic effects of Sb and Se. The lack of cytotoxicity from the unmodified fibres in our work, demonstrates the potential biocompatibility of medical devices developed using the Ge_20_Sb_10_Se_70_ at% glass composition.

## Funding

This work was supported by the Engineering and Physical Sciences Research Council (EPSRC) [*via* grant number EP/N50970X/1 and through the thematic programme: Wave Phenomena in Complex Media and EP/M008983/1].

## Conflicts of interest

There are no conflicts to declare.

## Supplementary Material

RA-011-D0RA00353K-s001
