# Supplementary material for: Evaluating the cytotoxicity of Ge–Sb–Se chalcogenide glass optical fibres on 3T3 mouse fibroblasts
Source: RSC Adv. 2021 Feb 25;11(15):8682–93. doi: 10.1039/d0ra00353k (PMC8695193; doi:10.1039/d0ra00353k)
Supplement: RA-011-D0RA00353K-s001 [file RA-011-D0RA00353K-s001.pdf]

ARTICLE

ELECTRONIC SUPPLEMENTARY INFORMATION

Received 00th January 20xx,
Accepted 00th January 20xx

DOI: 10.1039/x0xx00000x

Evaluating the cytotoxicity of Ge-Sb-Se chalcogenide glass optical fibres on 3T3 mouse fibroblasts.

David Mabwa*[a,b], Teo Kubiena [a,b], Harriet Parnell[a,b], Rong Su[c], David Furniss[a,b], Zhuoqi Tang[a,b], Richard Leach[c], Trevor M. Benson[a], Colin A. Scotchford[b], Angela B. Seddon*[a,b].

In vivo cancer detection based on the mid-infrared molecular fingerprint of tissue is promising for the fast diagnosis and treatment of suspected cancer patients. Few materials are mid-infrared transmissive, even fewer, which can be converted into functional, low-loss optical fibres for in vivo non-invasive testing. Chalcogenide-based glass optical fibres are, however, one of the few. These glasses are transmissive in the mid-infrared and are currently under development for use in molecular sensing devices. The cytotoxicity of these materials is however unknown. The cytotoxicity of Ge-Sb-Se chalcogenide optical glass fibres on 3T3 mouse fibroblast cells is here investigated. Fibres exposed to four different pre-treatment conditions are used: as-drawn (*AD*), propylamine-etched (*PE*), oxidised-and-washed (*OW*) and oxidised (*Ox*). To achieve the latter two conditions, fibres are treated with H_2O_2(aqueous (aq.)) and dried to produce a surface oxide layer; this is either washed off (*OW*) or left on the glass surface (*Ox*). Cellular response is investigated *via* 3-day elution and 14-day direct contact trials. The concentration of the metalloids (Ge, Sb and Se) in each leachate was measured *via* inductively coupled plasma mass spectrometry. Cell viability is assessed using the neutral red assay and scanning electron microscopy. The concentration of Ge, Sb and Se ions after a 3-day dissolution was as follows. In AD leachates, Ge: 0.40 mg/L, Sb: 0.17 mg/L, and Se: 0.06 mg/L. In PE leachates, Ge: 0.22 mg/L, Sb: 0.15 mg/L, and Se: 0.02 mg/L. In Ox leachates, Ge: 823.8 mg/L, Sb: 2586.6 mg/L, and Se: 3750 mg/L. Direct contact trials show confluent cell layers on *AD*, *PE* and *OW* fibres after 14-days, while no cells are observed on the Ox surfaces. A > 50 % cell viability is observed in *AD*, *PE* and *OW* eluates after 3-days, when compared with *Ox* eluates (< 10% cell viability). Toxicity in *Ox* is attributed to the notable pH change, from neutral pH 7.49 to acidic pH 2.44, that takes place on dissolution of the surface oxide layer in the growth media. We conclude, as-prepared Ge-Sb-Se glasses are cytocompatible and toxicity arises when an oxide layer is forced to develop on the glass surface.

1. Materials and Methods

1.1. Bulk Glass preform and Optical Fibre preparation

Antimony (Sb) (Cerac, 99.9999 % purity) and selenium (Se) (Materion, 99.999 % purity), purified by heating under 10^{-6} Pa, at 570 °C / 3 h and 260 °C / 0.5 h, respectively, were batched along with germanium (Ge) (Materion, <1 ppm OH, 99.999 % purity, as-received) into a silica glass ampoule (MultiLab, 10 mm / 14 mm = internal/external diameter, which had been prior rinsed in triplicate with deionised water then dried at ambient under ~10^{-1} Pa followed by purification by baking in air at 1 atm (1.013 x 10^5 Pa) / 900 °C / 6 h then vacuum: 10^{-6} Pa/ 900 °C / 6 h).

The $Ge_{20}Sb_{10}Se_{70}$ at. % glass batch was melted *in situ* inside the sealed, pre-purified silica glass ampoule at 900 °C / 12 h then the ampoule and contents were air-quenched to form a glass rod preform at the onset glass transition temperature (T_g), which for the as-batched composition $Ge_{20}Sb_{10}Se_{70}$ atomic % (at. %) was 214 ± 2 °C, from differential scanning calorimetry. Following this, the glass preform was annealed, *in situ* inside the same sealed, silica glass ampoule at T_g for 1 hour and finally cooled to ambient temperature to form a fibre optic preform (code: MDM223), which was 10 mm in diameter and 68 mm in length.

The fibre optic preform was removed from the silica glass ampoule and was fibre drawn in a He-purged, radio-frequency based furnace supported on an in-house customised, Heathway fibre-drawing tower. The tower was located within a 10,000-class cleanroom. The rod-preform was supported vertically on the tower, heated to a viscosity close to $10^{4.5}$ Pas and drawn into

[a] Mid-Infrared Photonics Group, George Green Institute for Electromagnetic Research Group, Faculty of Engineering, University of Nottingham, Nottingham NG7 2RD, UK.
[b] Advanced Materials Research Group, Faculty of Engineering, University of Nottingham, Nottingham NG7 2RD, UK.
[c] Manufacturing Metrology Team, Faculty of Engineering, University of Nottingham, Nottingham NG7 2RD, UK.
* Corresponding authors: david.mabwa@nottingham.ac.uk, angela.seddon@nottinghan.ac.uk
Electronic Supplementary Information (ESI) available: [details of any supplementary information available should be included here]. See DOI: 10.1039/x0xx00000x

Please do not adjust margins

52 m fibre (coded FDM093) with a diameter of 210 µm ± 20 µm and optical loss similar to the non-distilled fibre in [1]. Fibre was stored on a 1 m perimeter reel in the 10,000-class cleanroom for up to 90 days during the course of these experiments (similar compositions have been stored in our cleanroom without degradation for 5 years).

The diameter of the drawn fibre was achieved through the equation below, which gave the appropriate fibre-pull rate needed. For example, to draw the 10 mm diameter rod into the 210 µm diameter fibre, a preform-feed rate of 2 mm/min was used, requiring a fibre pull rate of 4.54 m/min.

$$Fibre\ pull\ rate\ (m/mm) = 0.001 \times (r_p^2/r_f^2) \times FR$$

where, r_p^2 is the radius of the preform (mm), r_f^2, the radius of the fibre (mm) and FR, the feed rate.

1.2. Physical analysis of $Ge_{20}Sb_{10}Se_{70}$ at. % glass optical fibres

1.2.1. Scanning electron microscopy

A Philips (FEI) XL30 scanning electron microscope (SEM) was used to assess the surface topography of $Ge_{20}Sb_{10}Se_{70}$ at. % glass optical fibre samples, before and after treatments. They were exposed to an accelerated voltage of 15 kV, and a maximum working distance of 10.9 mm was used.

1.2.2. Surface topography *via* coherent scanning interferometry

The surface texture of the $Ge_{20}Sb_{10}Se_{70}$ at. % glass optical fibre samples were measured using a Zygo NewView™ 8300 coherence scanning interferometry (CSI) system [2], with an objective magnification of 50× [numerical aperture (NA) of 0.55]. Two fibres from each pre-treatment condition were selected and the surface texture measured at ten different sections of each fibre, along the fibre longitudinal axis and at random points orthogonal to the fibre axis. Each measurement of texture had a field-of-view of approximately (170×170) µm and the distance between two adjacent measurements was 500 µm.

Surface data analysis was performed by means of MountainsMap® (Digital Surf). Areal texture parameter, *Sa*, was used to quantify the texture. *Sa* gives the arithmetical mean of the height deviation of each surface point over the measured area. The cylindrical form of the fibre was numerically removed and a high-pass Gaussian filter with cut-off spatial wavelength of 80 µm was applied to the surface before calculating *Sa* [3].

1.3. Cytotoxicity tests

1.3.1. An elute analysis: pH

The pH of AD, PE and O60 eluates was measured with an accuracy of ± 0.001 at ambient temperatures using the Jenway 3510 pH meter.

1.3.2. Cell-line

3T3 mouse fibroblast cells were used in this study (acquired from Public Health England, Cultures Collection, Porton Down, Salisbury, UK). Cell growth was initiated at passage 7 and grown to passage 9 before seeding. A 96-well plate (material: polystyrene, ThermoScientific), with an individual well area of 3.31 x 10^{-1} cm^{-2}, was used for both the direct contact and the elution trials. The seeding cell rate used for both types of trial was 15.5 x 10^3 cm^{-2}. Note that the growth was arrested for observation at days 1 and 3 for the elution experiments, and days 1, 3, 7 and 14 for the direct contact experiments.

1.3.3. Culture medium for 3T3 mouse fibroblast cells

To prepare the cell culture medium, 75 mg of ascorbic acid (Fisher Scientific, UK) was mixed with 50 ml of foetal bovine serum (FBS, Fisher Scientific, UK) and filtered through a 0.2 µm filter into 500 ml of Dulbeccos Modified Eagles Medium (DMEM) (Sigma Aldrich, UK). This filtrate was then further mixed with 5 ml of L-glutamine, 5 ml of non-essential amino acids (NEAA), 10 ml of antibiotics-antimycotics (AA/AM) and 10 ml of HEPES (hydroxyethyl-piperazineethanesulfonic acid) buffer (cell culture supplements from Gibco Invitrogen, UK) and frozen in 50 ml sterile universal tubes (material: polypropylene, Sigma Aldrich, for a maximum of 14 days). The culture medium was prepared under sterile conditions and ambient temperature in a class II microbiological cabinet [4]. This protocol is further described by Qiu *et al*. [5].

1.4. Statistical analysis of elution, direct contact and surface roughness analysis

Statistical analysis was performed on the results of the elution and direct contact trials and on the fibre surface texture results obtained from CSI measurements. The IBM SPSS Statistics 24 software was used to perform the statistical analysis. One-way ANOVA analysis was performed alongside multiple comparisons based on Tukey HSD, to determine significant differences [6]. A statistically significant difference, or result, was determined when the p-value (probability-value) was < 0.05. The sample repetition number (n) for each of the elution and direct contact trials was 5, and for the texture results obtained from CSI, n=10 for *AD*, and n=20 for *PE*, *OW*, *O10*, *O30*, and *O60*.

2. Results

2.1. Coherence scanning interferometry and scanning electron microscopy

2.1.1. AD, PE, OW, O10, O30, O60

As described in Section 1.2.2, the surface texture of each fibre sample was measured to an accuracy of ± 1 nm using coherence scanning interferometry. Two fibres from each pre-treatment conditions were selected, and ten texture measurements were then collected at different locations along the longitudinal axis of each fibre. The areal texture parameter (*Sa*) was used to quantify the surface texture.

As is seen in Figure 1 (A1-3), unmodified $Ge_{20}Sb_{10}Se_{70}$ at. % fibre possessed a comparatively smooth surface. Figure 1 (A1) shows a three-dimensional (3D) representation of the measured surface with few nanometre-sized flaws. The SEM images of *AD* fibre in Figure 1-A3 show features (ripple-like features with sizes ranging from 10 µm to 50 µm) observed on the fibre surface,

This journal is © The Royal Society of Chemistry 20xx

Please do not adjust margins

Please do not adjust margins

these were however infrequent. The average *Sa* calculated for *AD* fibre was 72 nm ± 25 nm, this was found to be substantially lower than the *Sa* of each of *PE*, *OW, O30* and *O60,* which exhibited *Sa* values of 169 nm ± 15 nm, 165 nm ± 53 nm, 161 nm ± 32 nm and 236 nm ± 84 nm, respectively. Hence, the AD fibre had the smoothest surface of all fibres. No statistically significant difference was found between the surface texture of *PE*, *OW* and *O30* fibres. On the other hand, the surface oxidised and successively solvent-washed to remove the oxide layer (*i.e.* the *OW* fibre) produced an even rougher surface in comparison to *AD* and *O10* (see Figure 1 (C1-3)).

As detailed in the main article, *O10*, *O30* and *O60* surfaces were prepared through submersion in H_2O_2 $_{(aq.)}$ for 10, 30 and 60 minutes, respectively, and allowed to air-dry for 24 hours.

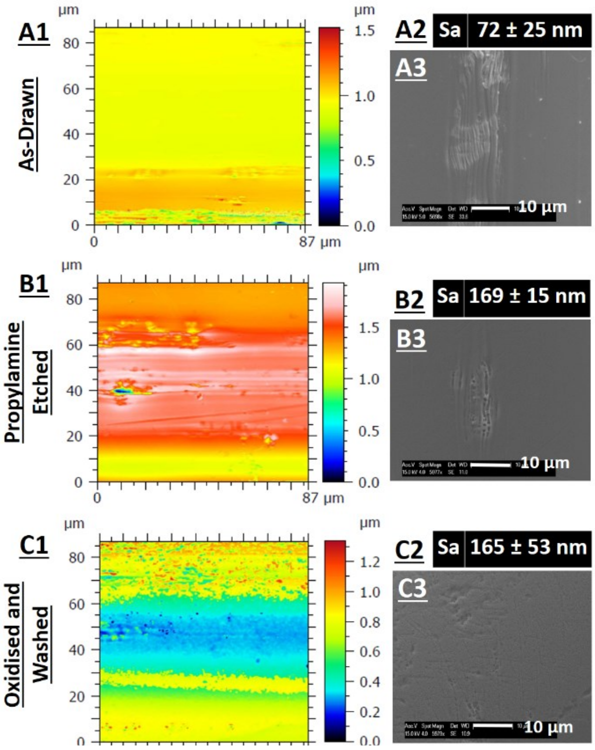

Figure 1. Surface topography of multiple $Ge_{20}Sb_{10}Se_{70}$ at. % fibre pieces subjected to different conditions to modify their texture. A1, B1 and C1 show the CSI measurements of the surface topographies of AD (As.D), PE (P.etch) and OW (Oxi-W), respectively. The average Sa value for each condition is shown in A2, B2, and C2, corresponding to AD, PE and OW, respectively. The variation (± X nm) of Sa for each surface was calculated via standard deviation. SEM images of AD, PE and OW are A3, B3 and C3, respectively. Significant differences were determined between AD and PE, OW, O30 (Oxi-10) and O60 (Oxi-60), between PE and both O10 and O60 and finally, between OW and both O10 and O60, using Tukey HSD, multiple comparisons, when p = < 0.05.

The resulting *Sa* values (see Figure 2 (A1-C3)) were found to increase with increasing time of exposure to the H_2O_2 $_{(aq.)}$ *viz: O10*, *O30* and *O60* surfaces were 81 nm ± 19 nm, 161 nm ± 32 nm and 236 nm ± 84 nm, respectively. A significantly higher *Sa* was obtained from *O60* in comparison to all other fibre surfaces produced in this work.

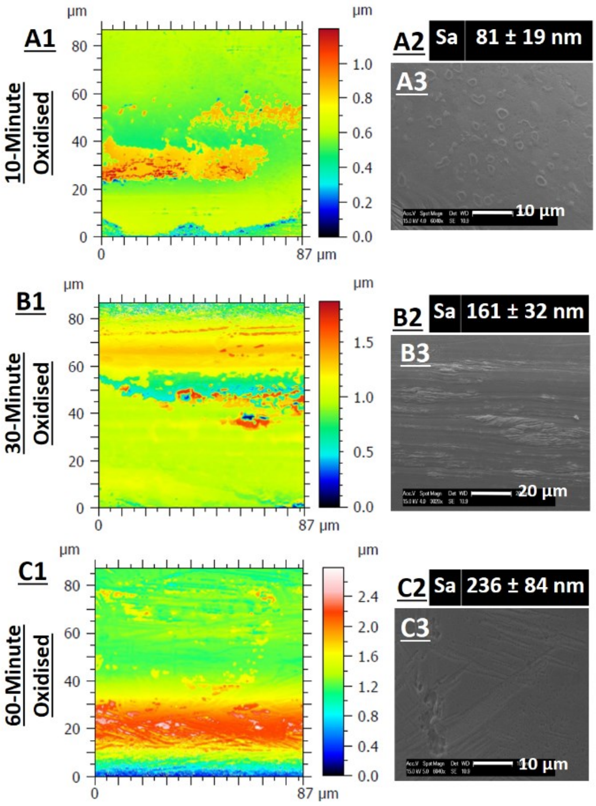

Figure 2. Surface topography of multiple $Ge_{20}Sb_{10}Se_{70}$ at. % fibre pieces subjected to different conditions to modify their texture. A1, B1 and C1 show the CSI measurements of the surface topographies of O10 (Oxi-10), O30 (Oxi-30) and O60 (Oxi-60), respectively. The average Sa value for each condition is shown in A2, B2, and C2, corresponding to O10, O30 and O60, respectively. The variation (± X nm) of Sa for each surface was calculated via standard deviation. SEM images of O10, O30 and O60 are A3, B3 and C3, respectively. Significant differences were determined between AD and PE, OW, O30 and O60, between PE and both O10 and O60 and finally, between OW and both O10 and O60, using Tukey HSD, multiple comparisons, when p = < 0.05.

2.2. pH Measurements

There was no notable difference between the pH of PE and AD eluates, when compared to deionised water (see Table 1). A distinct fall in pH is however observed with O60 eluates (2.44 – 2.11) compared to deionised water (7.49 – 7.31). The time for leeching to occur shows no significant influence on the pH.

Table 1. The pH of eluates (PE, AD, O60), from day 1 (D1) to, day 14 (D14) compared to deionised water.

	pH			
Pre-treatment	D1	D3	D7	D14
Deionised Water	7.49	7.31	7.34	7.49
PE	7.87	7.05	7.12	7.27
AD	7.84	7.06	7.14	7.25
O60	2.44	2.14	2.11	2.21

Please do not adjust margins

Please do not adjust margins

References

1 H. Parnell, D. Furniss, Z. Tang, Y. Fang, T. M. Benson, C. L. Canedy, C. S. Kim, M. Kim, C. D. Merritt, W. W. Bewley, I. Vurgaftman, J. R. Meyer and A. B. Seddon, "High purity Ge-Sb-Se/S step index optical fibres.," *Optical Materials Express,* vol. 9, no. 9, pp. 3616-3626, 2019.

2 P. de Groot and R. K. Leach, Chapter 9. Coherence scanning interferometry in optical measurment of surface topography, Springer, 2011.

3 ISO, "25178-2:2012 Geometric product specifications (GPS) - Surface texture: Areal - Part 2: Terms, definitions and surface texture parameters," International Organisation for Standards, 2012.

4 L. Cyster, L. France , J. Ratcliffe and M. Moles, "Cell culture training and protocols manual," University of Nottingham, Nottingham, 2011.

5 L. Qui, W. S. Lai, D. J. Stumpo and P. J. Blackshear, "Mouse embryonic fibroblast cell culture and stimulation," *Bio-protocol,* vol. 6, no. 13, 2016.

6 Y. Benjamini, H. Braun and W. John, "Tukey's contibutions to multiple comparisons," *The Annals of Statistics ,* vol. 30, no. 6, pp. 1576-94, 2002.

This journal is © The Royal Society of Chemistry 20xx

Please do not adjust margins
